# Prevalence and burden of illness of treated hemolytic neonatal hyperbilirubinemia in a privately insured population in the United States

**DOI:** 10.1186/s12887-019-1414-x

**Published:** 2019-02-11

**Authors:** Tzy-Chyi Yu, Chi Nguyen, Nancy Ruiz, Siting Zhou, Xian Zhang, Elaine A. Böing, Hiangkiat Tan

**Affiliations:** 1 Mallinckrodt Pharmaceuticals, Bedminster, NJ 07921 USA; 2 HealthCore, Inc., An Independent Subsidiary of Anthem, Inc, Wilmington, DE 19801 USA

**Keywords:** Hemolytic neonatal hyperbilirubinemia, Neonatal hyperbilirubinemia, Prevalence, Clinical characteristics, Healthcare resource utilization, Costs, Burden of illness

## Abstract

**Background:**

Prevalence of hemolytic neonatal hyperbilirubinemia (NHB) is not well characterized, and economic burden at the population level is poorly understood. This study evaluated the prevalence, clinical characteristics, and economic burden of hemolytic NHB newborns receiving treatment in U.S. real-world settings.

**Methods:**

This cohort study used administrative claims from 01/01/2011 to 08/31/2017. The treated cohort had hemolytic NHB diagnosis and received phototherapy, intravenous immunoglobulin, and/or exchange transfusions. They were matched with non-NHB newborns who had neither NHB nor related treatments on the following: delivery hospital/area, gender, delivery route, estimated gestational age (GA), health plan eligibility, and closest date of birth within 5 years. Inferential statistics were reported.

**Results:**

The annual NHB prevalence was 29.6 to 31.7%; hemolytic NHB, 1.8 to 2.4%; treated hemolytic NHB, 0.46 to 0.55%, between 2011 and 2016. The matched analysis included 1373 pairs ≥35 weeks GA. The treated hemolytic NHB cohort had significantly more birth trauma and hemorrhage (4.5% vs. 2.4%, *p* = 0.003), vacuum extractor affecting newborn (1.9% vs. 0.8%, *p* = 0.014), and polycythemia neonatorum (0.8% vs. 0%, *p* = 0.001) than the matched non-NHB cohort. The treated hemolytic NHB cohort also had significantly longer mean birth hospital stays (4.5 vs. 3.0 days, *p* < 0.001), higher level 2–4 neonatal intensive care admissions (15.7% vs. 2.4, 15.9% vs. 2.8 and 10.6% vs. 2.5%, respectively, all *p* < 0.001) and higher 30-day readmission (8.7% vs. 1.7%, *p* < 0.001).

One-month and one-year average total costs of care were significantly higher for the treated hemolytic NHB cohort vs. the matched non-NHB cohort, $14,405 vs. $5527 (*p* < 0.001) and $21,556 vs. $12,986 (*p* < 0.001), respectively. The average costs for 30-day readmission among newborns who readmitted were $13,593 for the treated hemolytic NHB cohort and $3638 for the matched non-NHB cohort, *p* < 0.001. The authors extrapolated GA-adjusted prevalence of treated hemolytic NHB in the U.S. newborn population ≥ 35 weeks GA and estimated an incremental healthcare expenditure of $177.0 million during the first month after birth in 2016.

**Conclusions:**

The prevalence of treated hemolytic NHB was 4.6–5.5 patients per 1000 newborns. This high-risk hemolytic NHB imposed substantial burdens of healthcare resource utilization and incremental costs on newborns, their caregivers, and the healthcare system.

## Background

Neonatal hyperbilirubinemia (NHB), a common condition in newborn infants, results from elevated blood bilirubin levels. The excessive bilirubin manifests as yellowing of the skin and the normally white outer layer of the eyeballs [1–3]. While most cases resolve quickly without intervention, NHB is a common reason for inpatient readmissions, and admission to the neonatal intensive care unit (NICU) [4, 5]. The prevalence of NHB is not precisely known, however, estimates suggest that approximately 50% full-term and 80% preterm [6] newborns develop some form of NHB. High-risk NHB occurs in 8–9% of neonates during the first week after birth [5, 7].

The origin of NHB may be physiologic or pathologic. Physiologic NHB may be caused by neonate immaturity and the resulting inability to cope with elevated levels of bilirubin [8]. This benign form resolves itself in 2–3 weeks following birth, and usually without treatment [1, 2]. Pathologic NHB may be caused by hemolytic disease of the newborn (HDN), red blood cell (RBC) enzyme deficiency, or impaired bilirubin excretion [9]. HDN results from incompatibilities between maternal and fetal blood types (Rh, ABO or a minor blood group), which may cause ruptures in fetal RBCs and elevated bilirubin levels. Hemolytic NHB usually appears within 24 h after birth [1, 2, 4].

The American Academy of Pediatrics (AAP) clinical practice guidelines address the assessment, screening, and treatment of NHB among infants at ≥35 weeks of gestation [10]. Risk assessment and treatment nomograms based on total serum bilirubin level, postnatal age in hours, and gestational age of the newborn with the presence or absence of risk factors are available to guide patient management [10]. Similar guidelines are not available for neonates at less than 35 weeks of gestation because of scant evidence-based data, differences in clinical manifestations and unclear treatment outcomes [11].

When treatment is indicated, AAP guidelines recommend phototherapy as the initial treatment [6, 10]. In cases where bilirubin levels continue to increase despite phototherapy, the guidelines recommend adding exchange transfusion of whole blood to the treatment regimen, typically in the NICU [2, 10]. For hemolytic cases, AAP guidelines recommend the administration of intravenous immunoglobulin (IVIg) as adjunctive therapy when bilirubin levels continue to rise despite intensive phototherapy [2, 10]. These challenges in management of high-risk hyperbilirubinemia substantially increase the urgency for safer and more effective screening and/or treatment options, especially when viewed against the knowledge that the permanent sequelae of kernicterus spectrum disorders (KSDs) might be prevented.

To the best of our knowledge, the prevalence of hemolytic NHB newborns receiving treatment has not been well characterized, and economic burden at the population level is poorly understood. Our study aimed to address this knowledge gap. We focused on newborns with hemolytic NHB who received treatment because the receipt of intervention indicated that those neonates met the AAP guideline for the recommendation of intervention in order to prevent severe NHB and the spectrum of associated complications [10, 12–14].

## Methods

### Design and data source

This retrospective matched cohort study used the HealthCore Integrated Research Database (HIRD^SM^), a geographically dispersed managed-care repository with claims data on more than 45 million enrollees residing across all 50 states, to identify infants born from 01/01/2011 through 08/31/2017. The HIRD is one of the largest privately insured population databases in the U.S [15]. This observational study was exempt from informed consent stipulations as researchers accessed a limited data set without individual enrollee identifiers and only summary statistics were reported. The study complied with all relevant provisions of the Health Insurance Portability and Accountability Act.

### Study population

Newborns were linked to their birth mothers via shared health plan subscriber identification (ID) numbers. Mothers’ delivery dates were verified within 32 days of newborns’ dates of birth using delivery codes (Appendix: Table 7). Infants with 30-day or longer continuous enrollment after birth and mothers with at least 12 months of continuous health plan enrollment before delivery were included. All newborns, regardless of their estimated gestational age (GA), were included for NHB prevalence estimation. The treated hemolytic NHB and matched non-NHB cohorts were selected among newborns ≥35 weeks GA. We excluded newborns < 35 weeks GA as there was no clinical practice guidelines available for this group due to lack of evidence-based data, variabilities in clinical manifestations, and uncertainties about treatment benefits [11].

NHB population was defined as newborns with ≥1 International Classification of Diseases (ICD)-9/10-CM diagnostic codes of NHB (ICD-9-CM = 773.0, 773.1, 773.2, 773.4, 774.x and ICD-10-CM = P55.x, P57.x, P58.x, P59.x) during the first 30 days after birth. Newborns with ≥1 ICD-9/10-CM diagnosis codes of ICD-9-CM = 773.0, 773.1, 773.2, 773.4, 774.0, 774.1, 774.7 and ICD-10-CM = P55.x, P57.x, P58.0, P58.1, P58.8, P58.9 were selected for the population of NHB with hemolysis indicators or hemolytic NHB (Appendix: Table 8).

#### Treated hemolytic NHB cohort

Treated hemolytic NHB cohort were selected from the hemolytic NHB population if they were ≥ 35 weeks GA and received at least one NHB intervention including: phototherapy (Healthcare Common Procedure Coding System [HCPCS] = E0202, S9098; ICD-9-CM procedure = 99.83; ICD-10-PCS = 6A600ZZ, 6A601ZZ), IVIg treatment along with NHB diagnosis code on the same claim (CPT = 90283, 90284; Generic Product Identifier [GPI] =19100020x; HCPCS = J1459, J1556, J1557, J1559, J1561, J1562, J1566, J1568, J1569, J1572, J1599), or exchange transfusions (CPT = 36450, 36456; ICD-9-CM procedure = 99.01; ICD-10-PCS = 30233H1, 30243H1).

#### Non-NHB cohort

A non-NHB cohort was established using 1:1 matching with newborns in the treated hemolytic NHB cohort ≥35 weeks GA. Inclusion in the non-NHB cohort required the absence of NHB diagnostic codes, no NHB treatment and a minimum of 30-day health plan enrollment after birth. Exact matching was performed based on delivery hospital/provider, gender, delivery route (C-section or vaginal), estimated GA, and post-index health plan continuous enrollment. When the matching of delivery hospital/provider was not possible, residence zip code (5-digit) was used instead. After all factors of interest were matched, newborns with the closest date of birth within 5 years were selected.

#### Gestational age

We calculated the GA of a newborn from prenatal procedure testing dates, from a range of common prenatal tests in the mother’s medical claims, using the weighted procedure date-based average methodology, as described by Wallace et al. [16]. This method demonstrated that 67% of all deliveries and 60% of preterm deliveries had estimated GA staying within one week of the actual GA [16].

### Outcomes

#### Prevalence of NHB

The annual prevalence of NHB, hemolytic NHB and treated hemolytic NHB were estimated for 2011 through 2016 as the number of newborns diagnosed with a disease divided by the total number of newborns after mother-infant linkage and health plan eligibility requirement during a particular calendar year.

#### Hospitalization and healthcare resource utilization

All-cause hospital measures included birth hospitalization, length of stay, NICU admissions, receipt of NHB treatments, and readmissions. The use of emergency department (ED) visits, physician office visits, other outpatient visits, and prescription fills were also presented. All healthcare resource utilization during the first 30 days and first year after birth were summarized.

#### Clinical characteristics and outcomes

The effects of hyperbilirubinemia on the brain and neurodevelopmental status were examined by evaluating occurrences of kernicterus, cerebral palsy, encephalopathy, hearing and vision loss, motor dysfunction, and neurodevelopmental delay during the first year after birth. These clinical outcomes were identified using ICD-9/10-CM diagnosis codes, requiring ≥1 diagnosis for inpatient/ED settings or ≥ 2 diagnoses on distinct dates for physician office settings (Appendix: Table 9).

#### Costs of care

Total all-cause costs during the first 30 days and first year after birth were reported. Since newborn care during birth hospitalization could be billed under their mothers’ plan ID, mothers’ delivery hospitalization costs were included to avoid any potential unequal underestimation between the newborn cohorts. These costs were the sum of the total paid amount by health plans, members’ out-of-pocket costs, and coordination of benefits. Total costs consisted of expenses incurred in inpatient, ED, office visits, other outpatient settings and pharmacy costs. Costs were adjusted for inflation using the Medical Care Consumer Price Index, and calculated in terms of 2017 U.S. dollars [17].

#### Extrapolation to the U.S. newborn population

Using U.S. Centers for Disease Control and Prevention (CDC) 2016 birth data by gestational age [18] and the estimated prevalence from our study, we applied a direct standardization method to extrapolate the 2016 U.S. GA-adjusted treated hemolytic NHB prevalence [19]. We then calculated population-level total healthcare expenditure based on our extrapolated prevalence and costs estimates.

### Statistical analysis

All outcome measures were compared between the treated hemolytic NHB and matched non-NHB cohorts. Statistical differences between groups were assessed using McNemar or McNemar-Bowker tests for categorical variables and paired t-tests or Wilcoxon signed-rank tests for continuous variables, respectively. A conventional alpha of 0.05 with two-tailed level of significance was used to interpret statistical significance. Statistical analyses were performed with SAS EG 7.1 (SAS Institute, Cary, NC).

## Results

### Study population

**Fig. 1:**
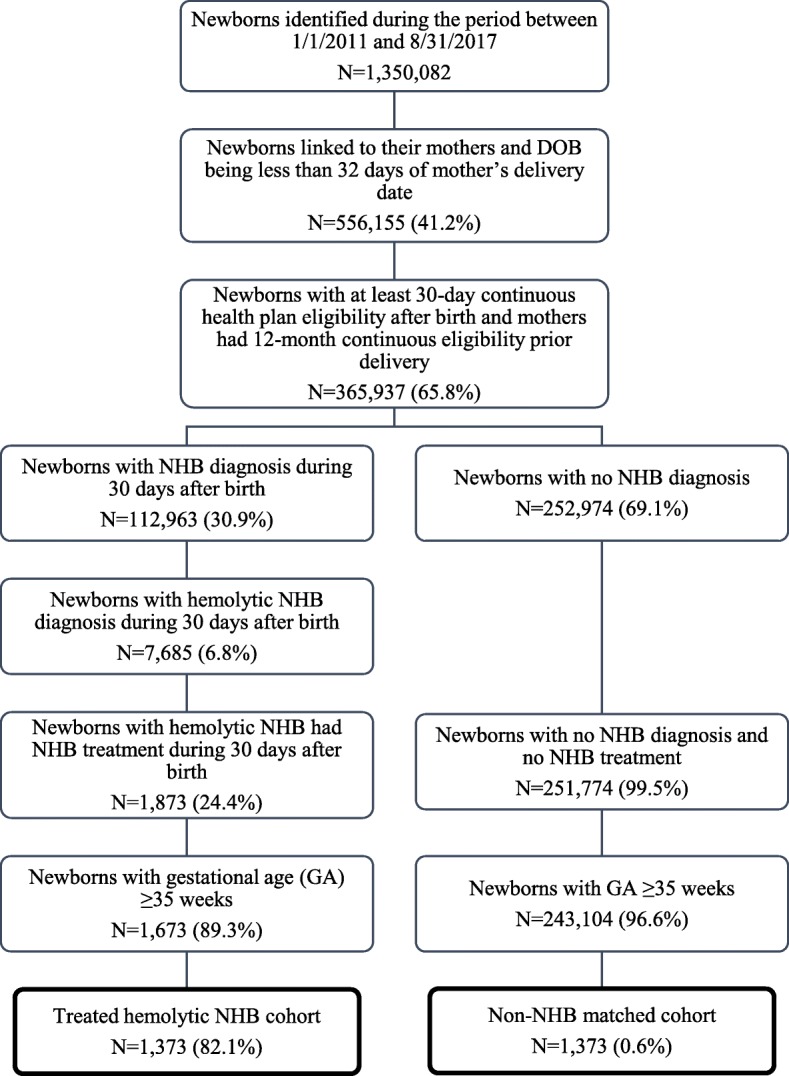
Flow chart of the study population. Treated hemolytic NHB newborns were exactly matched to non-NHB newborns on delivery hospital/provider, gender, delivery route (Csection or vaginal), estimated GA, and post-index health plan continuous enrollment. When the matching of delivery hospital/provider was not possible, residence zip code (5-digit) was used instead. After all of the above factors were matched, newborn with the closest DOB within 5 years was selected. DOB: date of birth; GA: gestational age; NHB: neonatal hyperbilirubinemia

Of the 1.4 million identified newborns, 365,937 were successfully linked to their birth mothers (Fig. 1). A total of 1673 newborns with hemolytic NHB received treatment and were of ≥35 weeks GA. Among those, 1373 treated hemolytic NHB newborns were matched with non-NHB newborns; the matching rate was 82.1%.

### Prevalence of NHB

**Fig. 2:**
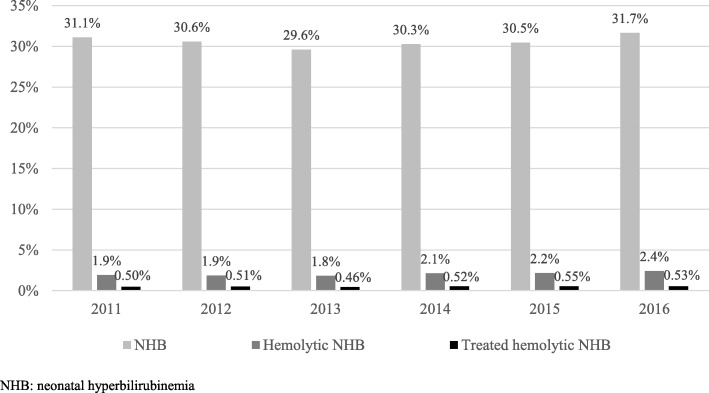
Prevalence of NHB, hemolytic NHB and treated hemolytic NHB stratified by GA from 2011 to 2016

The annual prevalence of NHB ranged from 29.6 to 31.7% during 2011 to 2016. The prevalence of hemolytic NHB during that period ranged from 1.8 to 2.4%, while the range for treated hemolytic NHB was 0.46 to 0.55% (Fig. 2). Upon stratification by estimated GA, the prevalence (95% Confidence Interval (CI)) of NHB among newborns < 35 weeks GA was 49.4% (95% CI: 48.6–50.1%), 38.4% (95% CI: 37.9–38.8%) of those 35–37 weeks GA, and 27.9% (95% CI: 27.7–28.1%) of those > 37 weeks GA during 2011 to 2016. Hemolytic NHB was reported in 2.8% (95% CI: 2.5–3.0%) of newborns < 35 weeks GA, 2.3% (95% CI: 2.1–2.4%) of those 35–37 weeks GA, and 2.0% (95% CI: 1.9–2.0%) of those > 37 weeks GA. The prevalence of treated hemolytic NHB among newborns < 35 weeks GA was 1.09% (95% CI: 0.93–1.25%), 0.70% (95% CI: 0.62–0.77%) of those 35–37 weeks GA, and 0.44% (95% CI: 0.41–0.46%) of those > 37 weeks GA (Table 1).

**Table 1:** Prevalence of NHB, hemolytic NHB and treated hemolytic NHB stratified by gestational age (GA) from 2011 to 2016

Estimated GA	Prevalence (95% Confidence Interval)		
	NHB	Hemolytic NHB	Treated hemolytic NHB
> 37 weeks GA	27.9% (27.7–28.1%)	2.0% (1.9–2.0%)	0.44% (0.41–0.46%)
35–37 weeks GA	38.4% (37.9–38.8%)	2.3% (2.1–2.4%)	0.70% (0.62–0.77%)
< 35 weeks GA	49.4% (48.6–50.1%)	2.8% (2.5–3.0%)	1.09% (0.93–1.25%)
All newborns	**30.6% (30.5–30.8%)**	**2.0% (2.0–2.1%)**	**0.51% (0.49–0.54%)**

*NHB* neonatal hyperbilirubinemia

### Mother and newborn demographic and clinical characteristics

The mean age of mothers of treated hemolytic NHB and matched non-NHB (32.2 vs. 32.1 years, *p* = 0.40), region of residence, type of health plan, comorbidity and gestational diabetes were similar at time of delivery. Slightly less than one-third (29.1%) of births was delivered by C-section, and 18.2% of newborns were of 35–37 weeks GA in each cohort (Table 2).

**Table 2:** Mother and newborn demographic and clinical characteristics

	Treated hemolytic NHB cohort (*N* = 1,373)	Matched non-NHB cohort (*N* = 1,373)	*p*-value^3^
Mothers			
Age on delivery (year), mean (SD)	32.2 (4.63)	32.1 (4.43)	0.401
Geographic region, *n* (%)			0.394
Northeast	289 (21.0)	285 (20.8)	
Midwest	503 (36.6)	494 (36.0)	
South	363 (26.4)	368 (26.8)	
West	212 (15.4)	218 (15.9)	
Other/Unknown^1^	6 (0.4)	8 (0.6)	
Health Plan type, *n* (%)			0.928
HMO	270 (19.7)	262 (19.1)	
PPO	831 (60.5)	845 (61.5)	
CDHP	272 (19.8)	266 (19.4)	
Modified Deyo-Charlson Comorbidity Index^2^, mean (SD)	0.1 (0.47)	0.1 (0.40)	0.274
Gestational diabetes, *n* (%)	239 (17.4)	222 (16.2)	0.367
C-section, *n* (%)	399 (29.1)	399 (29.1)	_
Newborns			
Gender, *n* (%)			_
Male	667 (48.6)	667 (48.6)	
Female	706 (51.4)	706 (51.4)	
Estimated gestational age, *n (%)*			_
35–37 weeks	250 (18.2)	250 (18.2)	
> 37 weeks	1,123 (81.8)	1,123 (81.8)	
Year of birth, *n* (%)			< 0.001
2011	217 (15.8)	217 (15.8)	
2012	211 (15.4)	195 (14.2)	
2013	187 (13.6)	206 (15.0)	
2014	197 (14.3)	239 (17.4)	
2015	228 (16.6)	239 (17.4)	
2016	208 (15.1)	188 (13.7)	
2017	125 (9.1)	89 (6.5)	

*NHB* neonatal hyperbilirubinemia, *SD* standard deviation, *HMO* Health Maintenance Organization, *PPO* Provider Preferred Organization, *CDHP* Consumer Driven Health Products

^1^Other/unknown region includes American Samoa, Guam, Northern Mariana Islands, Puerto Rico, Virgin Islands or unknown region

^2^Modified Deyo-Charlson Comorbidity Index was estimated using ICD-9/10-CM codes by Beyrer et al. [36]

^3^p-value calculated using McNemar test or McNemar-Bowker test for categorical variables and paired t-test or Wilcoxon signed-rank test for continuous variables

### NHB treatment

During birth hospitalizations, 69.1% of the treated hemolytic NHB cohort received treatment. During the first 30 days after birth, 98.9% received phototherapy only, 0.3% received exchange transfusion only, 0.1% received phototherapy plus IVIg, and 0.7% received phototherapy plus exchange transfusion (Table 3).

**Table 3:** NHB treatment pattern during 30 days after birth

Treatment pattern	Treated hemolytic NHB cohort (*N* = 1373)
During birth hospitalization (mutually exclusive)	
Any NHB treatment during birth hospitalization, *n*%	949 (69.1)
Phototherapy only, *n*%	937 (68.2)
IVIg only, *n*%	0 (0)
Exchange transfusion only, *n*%	4 (0.3)
Phototherapy + IVIg, *n*%	2 (0.1)
Phototherapy + Exchange transfusion, *n*%	6 (0.4)
IVIg + Exchange transfusion, *n*%	0 (0)
Phototherapy + IVIg + Exchange transfusion, *n*%	0 (0)
During 30 days after birth (mutually exclusive)	
Phototherapy only, *n*%	1358 (98.9)
IVIg only, *n*%	0 (0)
Exchange transfusion only, *n*%	4 (0.3)
Phototherapy + IVIg, *n*%	2 (0.1)
Phototherapy + Exchange transfusion, *n*%	9 (0.7)
IVIg + Exchange transfusion, *n*%	0 (0)
Phototherapy + IVIg + Exchange transfusion, *n*%	0 (0)

*NHB* neonatal hyperbilirubinemia, *IVIg* intravenous immunoglobulin

### Newborn clinical conditions and neurodevelopmental disorders

Newborns in the treated hemolytic NHB cohort had significantly higher proportions of birth trauma and hemorrhage (4.5% vs. 2.4%, *p* = 0.003), delivery by vacuum extractor affecting newborn (1.9% vs. 0.8%, *p* = 0.014), and polycythemia neonatorum (0.8% vs. 0.0%, *p* = 0.001) compared to the matched non-NHB cohort (Table 4). No difference was observed in neurodevelopmental disorders during the first year after birth between cohorts. Nine (1.2%) of the treated hemolytic NHB newborns had kernicterus.

**Table 4:** Newborn clinical conditions and neurodevelopmental disorders

	Treated hemolytic NHB cohort	Matched non-NHB cohort	*p*-value^1^
Clinical conditions during 30 days after birth, total *n*	1,373	1,373	
Breech delivery and extraction affecting fetus or newborn, *n* (%)	114 (8.3)	107 (7.8)	0.579
Birth trauma and hemorrhage, *n* (%)	62 (4.5)	33 (2.4)	0.003
Delivery by vacuum extractor affecting fetus or newborn, *n* (%)	26 (1.9)	11 (0.8)	0.014
Polycythemia neonatorum, *n* (%)	11 (0.8)	0 (0)	0.001
Other malpresentation, malposition, and disproportion during labor and delivery affecting fetus or newborn, *n* (%)	9 (0.7)	9 (0.7)	1.000
Forceps delivery affecting fetus or newborn, n (%)	5 (0.4)	7 (0.5)	0.564
Neonatal hematemesis and melena due to swallowed maternal blood, n (%)	0 (0)	0 (0)	_
Neurodevelopmental disorders during one year after birth, total n	**765**	**765**	
Kernicterus, *n* (%)	9 (1.2)	0 (0)	0.004
Motor dysfunction, *n* (%)	4 (0.5)	2 (0.3)	0.687
Hearing loss, *n* (%)	3 (0.4)	2 (0.3)	1.000
Encephalopathy, *n* (%)	2 (0.3)	2 (0.3)	1.000
Abnormal behavior, *n* (%)	1 (0.1)	3 (0.4)	0.625
Cerebral palsy, *n* (%)	1 (0.1)	0 (0)	1.000
Vision loss, *n* (%)	0 (0)	2 (0.3)	0.500
Neurodevelopmental delay, *n* (%)	0 (0)	1 (0.1)	1.000
Cognitive disorders, *n* (%)	0 (0)	0 (0)	_
Language disorders, *n* (%)	0 (0)	0 (0)	_

*NHB* neonatal hyperbilirubinemia

^1^*p*-values calculated using McNemar test or Fisher’s exact test for binary variables

### Healthcare resource utilization and costs during 30 days after birth

Treated hemolytic NHB newborns had longer average length of stay during birth hospitalization (4.5 days vs. 3.0 days; *p* < 0.001), and a greater proportion were admitted to NICU (82.6% vs. 70.0%; p < 0.001) compared to matched non-NHB newborns (Table 5). Significantly higher proportions of treated hemolytic NHB newborns were admitted to NICU levels 2–4 (15.7% vs. 2.4%; 15.9% vs. 2.8%; and 10.6% vs. 2.5%, respectively; all *p* < 0.001). Hospital readmissions and physician office visits were significantly higher for treated hemolytic NHB newborns than the matched non-NHB cohort, 8.7% vs. 1.7% (*p* < 0.001) and 90.8% vs. 82.6% (p < 0.001), respectively. No difference was reported for ED visits (1.7% vs. 1.4%, *p* = 0.54) and prescription fills (6.3% vs. 6.0%, *p* = 0.81) between the groups.

**Table 5:** Healthcare resource utilization and costs during 30 days after birth

	Treated hemolytic NHB cohort (*N* = 1373)	Matched non-NHB cohort (*N* = 1373)	*p*-value^1^
All-cause healthcare resource use			
Inpatient			
Birth hospitalization LOS, mean (SD)	4.5 (6.06)	3.0 (5.74)	< 0.001
NICU admission during birth hospitalization, *n* (%)	1,134 (82.6)	961 (70.0)	< 0.001
NICU Level 1	903 (65.8)	910 (66.3)	0.713
NICU Level 2	215 (15.7)	33 (2.4)	< 0.001
NICU Level 3	218 (15.9)	38 (2.8)	< 0.001
NICU Level 4	146 (10.6)	35 (2.5)	< 0.001
Readmission within 30-days after birth, *n* (%)	119 (8.7)	23 (1.7)	< 0.001
LOS, mean (SD)	2.4 (2.62)	1.7 (1.34)	0.033
Emergency room visits, *n* (%)	23 (1.7)	19 (1.4)	0.537
Number of visits, mean (SD)	1.0 (0.21)	1.1 (0.23)	0.919
Physician office visits, *n* (%)	1,247 (90.8)	1,134 (82.6)	< 0.001
Number of visits, mean (SD)	2.8 (1.49)	2.2 (1.15)	< 0.001
Other outpatient visits^2^, *n* (%)	1,001 (72.9)	427 (31.1)	< 0.001
Number of visits, mean (SD)	3.8 (3.29)	1.5 (1.26)	< 0.001
Prescription fills, *n* (%)	86 (6.3)	83 (6.0)	0.811
Number of fills, mean (SD)	1.2 (0.43)	1.1 (0.36)	0.533
All-cause healthcare costs, mean (SD), 2017 USD			
Medical costs	$14,403 ($43,918)	$5,524 ($50,078)	< 0.001
Inpatient (including birth hospitalization)	$13,794 ($43,949)	$5,216 ($50,083)	< 0.001
Birth hospitalization	$12,616 ($42,475)	$5,155 ($50,080)	< 0.001
Readmission during 30 days after birth^3^	$13,593 ($34,524)	$3,638 ($5685)	< 0.001
Emergency department	$20 ($187)	$17 ($169)	0.636
Physician office visit	$313 ($258)	$224 ($203)	< 0.001
Other outpatient visits	$276 ($651)	$67 ($289)	< 0.001
Pharmacy costs	$2 ($12)	$2 ($28)	0.923
Total medical and pharmacy costs	**$14,405 ($43,918)**	**$5,527 ($50,079)**	**< 0.001**
Incremental all-cause healthcare costs			
Treated hemolytic NHB newborn incremental costs	$8,878 ($59,943)		
Mother’s delivery incremental costs^4^	$503 ($19,969)		
Total incremental costs	**$9,381 ($63,558)**		

*NHB* neonatal hyperbilirubinemia, *SD* standard deviation, *LOS* length of stay

^1^p-values calculated using McNemar test for binary variables and Wilcoxon signed-rank test for continuous variables

^2^Other outpatient visits included durable medical equipment, imaging, medication & related services, procedures, physician other services, tests and occupational, physical or speech therapy

^3^Readmission costs calculated among those who had readmission during the first 30 days after birth, including 119 newborns in treated hemolytic NHB cohort and 23 newborns in matched non-NHB cohort

^4^A newborn’s care and treatment could be billed to his/her mother’s plan during birth hospitalization; mother’s incremental costs of delivery hospitalization were included

Mean (SD) total 30-day all-cause costs for the newborns were $14,405 ($43,918) for the treated hemolytic NHB group and $5,527 ($50,079) for the matched non-NHB cohort (p < 0.001). The treated hemolytic NHB group incurred mean (SD) total inpatient hospitalization costs of $13,794 ($43,949) compared to $5,216 ($50,083) in the matched non-NHB group, *p* < 0.001. The average costs of readmissions among those readmitted to the hospitals were $13,593 ($34,524) and $3,638 ($5,685) for the treated hemolytic NHB and non-NHB groups, respectively. The mean (SD) 30-day incremental total all-cause costs associated with treated hemolytic NHB newborns was $9,381 ($63,558) composed of $8,878 ($59,943) from newborns plus $503 ($19,969) from mothers’ delivery hospitalization.

### Healthcare resource utilization and costs during one year after birth

Of 1,373 pairs, 765 (55.7%) matched pairs with one-year follow-up were included in the analysis. There was no statistically significant difference between the two cohorts in inpatient admissions and ED visits during the period from 31 days to 1 year after birth. Physician office visits and prescription fills were slightly higher in the treated hemolytic NHB group compared to the matched non-NHB group (99.7% vs. 97.4%, *p* < 0.001 and 69.7% vs. 63.5%, *p* = 0.009, respectively). The mean (SD) total one-year all-cause costs incurred by the treated hemolytic NHB cohort were $21,556 ($60,823) compared to $12,986 ($72,164) in the matched non-NHB cohort, *p* < 0.001. The average (SD) one-year incremental total all-cause costs associated with treated hemolytic NHB was $9,383 ($84,478), consisting of $813 ($12,922) from mother’s delivery hospitalization and $8,570 ($82,379) from newborns (Table 6).

**Table 6:** Healthcare resource utilization and costs during one year after birth

	Treated hemolytic NHB cohort (*N* = 765)	Matched non-NHB cohort (*N* = 765)	*p*-value^1^
All-cause healthcare resource use			
Inpatient			
Readmission within 30-days after birth, *n* (%)	60 (7.8)	14 (1.8)	< 0.001
Inpatient admission from 31 days to one year after birth	36 (4.7)	24 (3.1)	0.109
Emergency room visits, *n* (%)	138 (18.0)	125 (16.3)	0.378
Number of visits, mean (SD)	1.3 (0.72)	1.2 (0.50)	0.690
Physician office visits, *n* (%)	763 (99.7)	745 (97.4)	< 0.001
Number of visits, mean (SD)	12.2 (5.44)	10.7 (4.92)	< 0.001
Other outpatient visits^2^, *n* (%)	763 (99.7)	736 (96.2)	< 0.001
Number of visits, mean (SD)	11.3 (7.25)	8.3 (7.28)	< 0.001
Prescription fills, *n* (%)	533 (69.7)	486 (63.5)	0.009
Number of fills, mean (SD)	4.3 (4.36)	3.9 (3.94)	0.140
All-cause healthcare costs, mean (SD), 2017 USD			
Newborns			
Medical costs	$21,407 ($60,808)	$12,784 ($71,669)	< 0.001
Inpatient (including birth hospitalization)	$16,679 ($58,723)	$8865 ($70,060)	< 0.001
Emergency department	$279 ($850)	$235 ($749)	0.199
Physician office visits	$1,443 ($847)	$1,248 ($864)	< 0.001
Other outpatient visits	$3,006 ($4,096)	$2,436 ($3,712)	< 0.001
Pharmacy costs	$149 ($359)	$202 ($1494)	0.038
Total newborn medical and pharmacy costs	**$21,556 ($60,823)**	**$12,986 ($72,164)**	**< 0.001**
Incremental all-cause healthcare costs			
Treated hemolytic NHB newborn incremental costs	$8,570 ($82,379)		
Mother’s delivery incremental costs^3^	$813 ($12,922)		
Total incremental costs	**$9,383 ($84,478)**		

*NHB* neonatal hyperbilirubinemia, *SD* standard deviation

^1^*p*-values calculated using McNemar test for binary variables and Wilcoxon signed-rank test for continuous variables

^2^Other outpatient visits included durable medical equipment, imaging, medication & related services, procedures, physician other services, lab tests and occupational, physical or speech therapy

^3^A newborn’s care and treatment could be billed to his/her mother’s plan during birth hospitalization; mother’s incremental costs of delivery hospitalization were included

### Extrapolation to the U.S. population

The extrapolation of 2016 U.S. GA-adjusted treated hemolytic NHB prevalence was 0.53%, 20,854 newborns (95% CI, 18,398-23,311) among 3.9 million newborns in the U.S. in 2016. Among newborns ≥35 weeks GA, the GA-adjusted prevalence of treated hemolytic NHB was 0.50% resulting in 18,872 newborns (95% CI, 16,523 - 21,221). The 18,872 treated hemolytic NHB newborns represent an estimated total healthcare expenditure of $271.9 million and incremental costs of $177.0 million compared with their counterparts without NHB during the first month after birth in the U.S. in 2016.

## Discussion

To the best of our knowledge, this is the first study to estimate the prevalence of high-risk hemolytic NHB newborns receiving intervention, and to quantify the burden of hemolytic NHB in the US. The proportions of newborns with hemolytic NHB who received treatment were 0.46 to 0.55% in a privately insured population in the US. Although not prevalent, those high-risk hemolytic NHB neonates who received treatment were associated with substantial healthcare resource utilization and incremental economic burden.

NHB research in the U.S. has been limited, and prevalence estimates vary markedly in the handful of studies in the literature. In a systematic review that included 14 studies to examine the effects and outcomes of phototherapy, Woodgate and Jardine noted that about 50% of full-term and 80% preterm newborns developed jaundice [6]. In a survey at medical centers that practiced universal pre-discharge total serum bilirubin (TSB) screening, Bhutani et al. reported jaundice in 84% of healthy newborns ≥35 weeks GA [20]. Another study, which used inpatient data from the Healthcare Costs and Utilization Project (HCUP), reported 15.6% of newborns had jaundice [21]. These variations could, in part, be due to differences in the study population, case definitions (e.g., TSB level vs. visible jaundice), data sources, and underdiagnosis or underreporting of mild cases. Mild NHB typically resolves without intervention, and may not be fully captured in administrative claims (used in our study) and hospital discharge data (HCUP). Such cases may not be reflected in reimbursements because of bundled payments, which could result in an underestimation of general NHB prevalence.

Our study focused on NHB specifically with etiology of hemolytic diseases, and we found that approximately 7% of the NHB cases were hemolytic NHB. Our estimated prevalence of treated hemolytic NHB (ranging from 0.46–0.55%) was comparable to < 1% of significant hemolysis reported by Wagle and Deshpande [22]. Chang et al. estimated that about 6% of newborns ≥35 weeks GA received phototherapy at Kaiser Permanente hospitals [23]. Using our estimate that 7% of the NHB newborns in this study had hemolytic NHB along with the assumption that all the newborns in the Chang et al. study had NHB, we inferred that approximately 0.42% of newborns in Chang et al. were phototherapy-treated hemolytic NHB — which is close to our estimate. Treatment rates could vary remarkably as treatment practice across hospitals/institutions differ in how cases are identified and when treatment should be initiated [24, 25]. Additionally, prior literature suggested that NHB patients could be under-treated. One U.S. study showed that only approximately half (54%) of healthy term newborns for whom AAP clinical practice guidelines recommended phototherapy received treatment [26].

We found that treated hemolytic NHB newborns had significantly longer length of stay during their birth hospitalization, higher 30-day readmission rates, higher NICU use and slightly higher rates of physician office visits, compared to their matched counterparts. Length of stay of mothers’ delivery hospitalizations were also slightly longer in the treated hemolytic NHB cohort (2.9 days vs 2.5 days, data not shown). These findings suggest significant burden to patients, their caregivers, and the healthcare system. Prior studies have shown that NHB was as major cause of readmission. Approximately half (51%) of all readmissions occurring 2 weeks after birth were attributable to NHB [27]. The increase in physician office visits we reported was also consistent with available literature, which found that NHB was associated with increased parental awareness, and newborns receiving phototherapy had higher rates of outpatient visits [28].

We also found that hemolytic NHB newborns who received treatment incurred 2.6 times the average costs of their matched non-NHB counterparts during the first 30 days after birth. The majority of the incremental cost was derived from birth hospitalizations. Indirect costs associated with patients’ and caregivers’ quality of life as well as caregivers’ loss of productivity could not be evaluated using claims data. As of now, no prior study has examined the economic burden of hemolytic NHB. One earlier study estimated the average cost of childbirth via vaginal or caesarian at $18,329 or $27,866, respectively, in a private health plan [29]. Those estimates were close to the average costs, $20,568, of the sum of maternal delivery (mean (SD) = $15,413 ($20,010), data not shown) and newborn birth hospitalization ($5,155 ($50,080), Table 5) in the non-NHB cohort in our study. Such comparability might warrant the representativeness and generalizability of our study results to other privately insured populations. In this study, we found that the majority of treated hemolytic NHB newborns received phototherapy. A total of 15 (1%) newborns received IVIg or ET, which are recommended by AAP when bilirubin levels continue to rise despite intensive phototherapy. This group imposed even greater economic burden with average (SD) total one-month all-cause costs of $81,065 ($133,767) (data not shown).

We extrapolated our findings to the entire U.S. newborn population in 2016. The extrapolation estimated total healthcare expenditure of $271.9 million and incremental costs of $177.0 million among 18,872 treated hemolytic NHB newborns as compared with their counterparts without NHB during the first month after birth. Our extrapolation assumed our estimates were applicable to the U.S. newborn population mainly insured by private insurance plans or Medicaid. This projection should be interpreted with caution as privately insured populations tend to have higher socioeconomic status and healthcare expenditures than the Medicaid population [30]. Further research in the Medicaid newborn population is warranted to examine our assumptions and estimates.

We did not observe significant difference in neurodevelopment delay, language disorders, motor dysfunction, cerebral palsy, abnormal behavior, encephalopathy, hearing and vision loss between treated hemolytic NHB newborns and the matched non-NHB cohort during the first year of birth. However, the observation period was likely too short as many of these conditions might not be identifiable nor noticeable in the first year of life. Kernicterus, a brain injury resulting from severe NHB, was found in nine newborns, approximately 1.2% of all treated hemolytic NHB newborns during the one-year follow up. Kernicterus has been reported from 1.0 to 3.7 cases per 100,000 live birth in the literature [31, 32], but these incidence rates were estimated for the general population in contrast to the high-risk hemolytic NHB population (treated) in this study. As hemolytic NHB was strongly correlated with higher incidences of birth trauma, polycythemia, and other subsequent morbidities which could also cause neurodevelopment disorders, neurodevelopment disorders in this population could be due to a combination of hemolytic NHB and other morbidities, rather than hemolytic NHB alone.

Effective management of high-risk hemolytic NHB is critical to reduce the impact of disease burden on patients, their caregivers, and the healthcare system. Several studies have investigated comprehensive approaches, such as pre-discharge bilirubin screening for all newborns [25], or the implementation of a standard pathway including treatment algorithms (e.g., requiring irradiance compliance to ensure consistent delivery of effective phototherapy) and education to increase awareness among clinicians [33]. These comprehensive approaches have demonstrated success in reducing costs, length of stay [33] and hospital readmission rates [25]. In addition, new treatment options are needed. For example, an investigational treatment – stannsoporfin (SnMP, a heme oxygenase inhibitor) with or without phototherapy was studied for use in the management of NHB or hemolytic NHB [34, 35].

### Limitations

Our results should be interpreted in light of certain limitations. Known risk factors such as family history, race and ethnicity, and breastfeeding status are not available in administrative claims data. Cases of mild NHB do not usually require intervention, and can be underdiagnosed and/or under-coded in administrative data leading to underestimation of NHB and hemolytic NHB. The use of phototherapy during hospitalization might not have been observed due to bundled payments and/or under-coding. Duration on phototherapy was also not captured. This study population was from a U.S. privately insured population, which may limit the generalizability of these results to other population segments, such as Medicaid.

## Conclusions

This is likely the first study estimating the prevalence of newborns with hemolytic NHB who received intervention in the U.S. This high-risk population imposes a substantial burden of healthcare resource utilization and incremental costs on newborns, their caregivers, and the healthcare system. Effective management protocols and emerging new treatments may help to mitigate the overall burden of hemolytic NHB.

## Appendix

**Table 7:** Codes to identify delivery

1. Codes to identify all deliveries	
CPT codes	
Routine obstetric care including antepartum care, vaginal delivery (with or without episiotomy, and/or forceps) and postpartum care	59400
Vaginal delivery only (with or without episiotomy and/or forceps)	59409
Vaginal delivery only (with or without episiotomy and/or forceps), including postpartum care	54910
External cephalic version, with or without tocolysis	59412
Delivery of placenta (separate procedure)	59414
Antepartum care only; 4–6 visits	59425
Antepartum care only; 7+ visits	59,426
Routine obstetric care including antepartum care, vaginal delivery (with or without episiotomy, and/or forceps) and postpartum care, after previous cesarean delivery	59610
Vaginal delivery only, after previous cesarean delivery (with or without episiotomy and/or forceps);	59612
Vaginal delivery only, after previous cesarean delivery (with or without episiotomy and/or forceps); including postpartum care	59614
Routine obstetric care including antepartum care, cesarean delivery, and postpartum care	59510
Cesarean delivery only	59514
Cesarean delivery only; including postpartum care	59515
Routine obstetric care including antepartum care, cesarean delivery, and postpartum care, following attempted vaginal delivery after previous cesarean delivery	59618
Cesarean delivery only, following attempted vaginal delivery after previous cesarean delivery;	59620
Cesarean delivery only, following attempted vaginal delivery after previous cesarean delivery; including postpartum care	59622
Revenue Codes	
Labor Room/Delivery	72.x
ICD-9-CM diagnosis	
Outcome of delivery	
Single liveborn	V27.0x
Twins, both liveborn	V27.2x
Twins, one liveborn one stillborn	V27.3x
Other multiple, all liveborn	V27.5x
Other multiple, some liveborn	V27.6x
Unspecified	V27.9x
Liveborn infants consuming healthcare	
Single liveborn	V30.xx
Twin, mate liveborn	V31.xx
Twin, mate stillborn	V32.xx
Twin, unspecified	V33.xx
Other multiple, mates all liveborn	V34.xx
Other multiple, mates all stillborn	V36.xx
Other multiple, unspecified	V37.xx
Unspecified	V39.xx
Normal delivery	650.xx
Forceps or vacuum extractor delivery without mention of indication	669.5x
Breech extraction, without mention of indication	669.6x
Cesarean delivery, without mention of indication	669.7x
ICD-9-CM procedure	
Forceps, vacuum, and breech delivery	72.xx
Other procedures inducing or assisting delivery	73.xx
Cesarean section and removal of fetus	74.xx
ICD-10-CM diagnosis	
Outcome of delivery	
Single liveborn	Z37.0x
Twins, both liveborn	Z37.2x
Twins, one liveborn one stillborn	Z37.3x
Other multiple, all liveborn	Z37.5x
Other multiple, some liveborn	Z37.6x
Unspecified	Z37.9x
Liveborn infants consuming healthcare	
Single liveborn	Z38.0x-Z38.2x
Twin liveborn	Z38.3x-Z38.5x
Other multiple liveborn	Z38.6x-Z38.8x
Encounter for full-term uncomplicated delivery	O80x
Encounter for cesarean delivery without indication	O82x
ICD-10-PCS procedure	
Extraction of POC, Classical, Open Approach	10D00Z0
Extraction of POC, Low Cervical, Open Approach	10D00Z1
Extraction of POC, Extraperitoneal, Open Approach	10D00Z2
Extraction of POC, Low Forceps, Via Opening	10D07Z3
Extraction of POC, Mid Forceps, Via Opening	10D07Z4
Extraction of POC, High Forceps, Via Opening	10D07Z5
Extraction of Products of Conception, Vacuum, Via Opening	10D07Z6
Extraction of POC, Int Version, Via Opening	10D07Z7
Extraction of Products of Conception, Other, Via Opening	10D07Z8
Delivery of Products of Conception, External Approach	10E0XZZ
2. Codes to identify cesarean delivery	
CPT codes	
Routine obstetric care including antepartum care, cesarean delivery, and postpartum care	59510
Cesarean delivery only	59514
Cesarean delivery only; including postpartum care	59515
Routine obstetric care including antepartum care, cesarean delivery, and postpartum care, following attempted vaginal delivery after previous cesarean delivery	59618
Cesarean delivery only, following attempted vaginal delivery after previous cesarean delivery;	59620
Cesarean delivery only, following attempted vaginal delivery after previous cesarean delivery; including postpartum care	59622
ICD-9-CM diagnosis	
Single liveborn, born in hospital, delivered by cesarean delivery	V30.01
Twin, mate liveborn, born in hospital, delivered by cesarean delivery	V31.01
Twin, mate stillborn, born in hospital, delivered by cesarean delivery	V32.01
Twin, unspecified whether mate stillborn or liveborn, born in hospital, delivered by cesarean delivery	V33.01
Other multiple, mates all liveborn, born in hospital, delivered by cesarean delivery	V34.01
Other multiple, mates all stillborn, born in hospital, delivered by cesarean delivery	V35.01
Other multiple, mates liveborn and stillborn, born in hospital, delivered by cesarean delivery	V36.01
Other multiple, unspecified whether mates stillborn or liveborn, born in hospital, delivered by cesarean delivery	V37.01
Liveborn infant, unspecified whether single, twin, or multiple, born in hospital, delivered by cesarean	V39.01
Cesarean delivery, without mention of indication	669.7x
ICD-9-CM procedure	
Cesarean section and removal of fetus	74.xx
ICD-10-CM diagnosis	
Single liveborn infant, delivered by cesarean	Z38.01
Twin liveborn infant, delivered by cesarean	Z38.31
Triplet liveborn infant, delivered by cesarean	Z38.62
Quadruplet liveborn infant, delivered by cesarean	Z38.64
Quintuplet liveborn infant, delivered by cesarean	Z38.66
Other multiple liveborn infant, delivered by cesarean	Z38.69
Encounter for cesarean delivery without indication	O82x
ICD-10-PCS procedure	
Extraction of POC, Classical, Open Approach	10D00Z0
Extraction of POC, Low Cervical, Open Approach	10D00Z1
Extraction of POC, Extraperitoneal, Open Approach	10D00Z2

**Table 8:** Codes to identify neonatal hyperbilirubinemia

Description	ICD-9-CM	ICD-10-CM
Hemolytic disease of fetus or newborn due to Rh isoimmunization	773.0	P55.0
Hemolytic disease of fetus or newborn due to ABO isoimmunization	773.1	P55.1
Hemolytic disease of fetus or newborn due to other and unspecified isoimmunization	773.2	P55.8, P55.9
Kernicterus of fetus or newborn due to isoimmunization	773.4	P57.0
Perinatal jaundice from hereditary hemolytic anemias	774.0	P58.8
Perinatal jaundice from other excessive hemolysis	774.1	P58.0, P58.2, P58.3, P58.41, P58.5, P58.8
Kernicterus of fetus or newborn not due to isoimmunization	774.7	P57.8
Rh isoimmunization of newborn	794	P55.0
ABO isoimmunization of newborn	794	P55.1
Other hemolytic diseases of newborn	793	P55.8
Hemolytic disease of newborn, unspecified	793	P55.9
Kernicterus due to isoimmunization	793	P57.0
Other specified kernicterus	793	P57.8
Kernicterus, unspecified	793	P57.9
Neonatal jaundice due to bruising	794	P58.0
Neonatal jaundice due to bleeding	794	P58.1
Neonatal jaundice due to other specified excessive hemolysis	794	P58.8
Neonatal jaundice due to excessive hemolysis, unspecified	794	P58.9

**Table 9:** Codes to identify NICU admission, gestational age estimation and newborn clinical conditions

Descriptions	ICD-9-CM	ICD-10-CM	CPT/ Revenue codes
Neonatal Intensive Care Unit (NICU)			
Level 1			Revenue: 171
Level 2			Revenue: 172
Level 3			Revenue: 173
Level 4			Revenue: 174
Prenatal tests and preterm codes for gestational age estimation			
Oral glucose tolerance test (OGTT)			82950, 82951, 82952
Alpha-fetoprotein (AFP)			82105
Inhibin-A (IHA)			86336
Ultrasound, pregnant uterus, real time with image documentation, fetal and maternal evaluation plus detailed fetal anatomic examination			76801,76802,76805,76810, 76811,76812,76813,76814, 76815,76816,76817,76818, 76819,76820
Pregnancy-associated protein plasma-A (PAPP-A)			84163
Chorionic villus sampling			59015
Preterm 35–37 weeks	765.28	P07.38, P07.39	
Preterm < 35 weeks	765.21, 765.22, 765.23, 765.24, 765.25,765.26, 765.27	P07.21, P07.22, P07.23, P07.24, P07.25, P07.26, P07.31, P07.32, P07.33, P07.34, P07.35, P07.36, P07.37	
Preterm unspecified	765.20,	P07.20, P07.30,	
		P07.0x, P07.1x,	
	644.2x	O60.1x	
Newborn clinical conditions			
Birth trauma and/or hemorrhage (associated with the development of potential jaundice from bleeding)	864.00–864.05,864.09, 865.00–865.04,865.09, 767.0,767.11,770.3, 772.10–772.14, 772.2–772.9	P10x,P12.0x,P12.1x, P12.3x,P15.0x, P15.1x,P26x, P52x, P53x, P54x	
Polycythemia neonatorum	776.4	P61.1x	
Neonatal hematemesis and melena due to swallowed maternal blood	777.3	P78.2x	
Breech delivery and extraction affecting fetus or newborn	763.0	P03.0x	
Forceps delivery affecting fetus or newborn	763.2	P03.2x	
Delivery by vacuum extractor affecting fetus or newborn	763.3	P03.3x	
Other malpresentation, malposition, and disproportion during labor and delivery affecting fetus or newborn	763.1	P03.1x	
Newborn neurodevelopmental disorders			
Developmental delay	299.xx, 315.8, 315.9, 330.8	F84x, F88x, F89x	
Cognitive disorders (nonspecific)	310.9x	F09x	
Language disorder (or speed disorder, dysarthria)	315.3x	F80x	
Motion dysfunction	315.4x, 781.2, 719.7, 781.3,	F82x, R26x, R27x	
Cerebral palsy	343.x, 333.71,	G80x	
Abnormal behavior	314.x, 312.x, 309.21, 313.x, 307.x,	F90x-F98x	
Bilirubin encephalopathy (kernicterus spectrum disorder)	773.4, 774.7	P57x	
Encephalopathy	348.3x	G93.4x	
Hearing loss	389.xx, 388.01, 388.2388.11	H90x, H91x	
Vision loss	368.xx	H53x	

## Abbreviations

AAP: American Academy of Pediatrics
CI: Confidence Interval
GA: Gestational age
HCRU: Healthcare resource utilization
HDN: Hemolytic disease of the newborn
ICD-9/10-CM: The international classification of diseases, 9/10th Revision, clinical modification
ICD-9-CM/10-PCS: The international classification of diseases, 9/10th Revision, procedure coding system
IVIg: Intravenous immunoglobulin
KSDs: Kernicterus spectrum disorders
NHB: Neonatal hyperbilirubinemia
NICU: Neonatal intensive care unit
RBC: Red blood cell
SD: Standard deviation
TSB: Total serum bilirubin
